# Effect of Intensity Modulated Radiotherapy (IMRT) on the immunity, physical status and clinical effect of locally advanced NSCLC patients

**DOI:** 10.12669/pjms.37.5.4188

**Published:** 2021

**Authors:** Jun-kai Xu

**Affiliations:** Jun-kai Xu Department of Radiotherapy, The Affiliated Hospital (Group) of Putian University, Putian, 351100, P.R. China

**Keywords:** IMRT, Camrelizumab, locally advanced, NSCLC, treatment

## Abstract

**Objectives:**

To evaluate the clinical value of radiotherapy combined with Camrelizumab in treating locally advanced non-small cell lung cancer (NSCLC) patients.

**Methods:**

80 locally advanced NSCLC patients were randomly divided into two groups (n=40). The control group was administered with intensity modulated radiation therapy (IMRT), whereas the experimental group with Camrelizumab in addition to IMRT. All the patients underwent clinical efficacy evaluation in terms of adverse drug reaction (ADR), physical status improvement after the treatment, and changes in T lymphocyte subpopulations (incl. CD3^+^, CD4^+^, CD8^+^, CD4^+^/CD8^+^).

**Results:**

The efficacy was found to be 70% and 47.5 in experimental group and control group, respectively, with the former being significantly better than the latter (p=0.03). The ADR rates were 50% and 37.5% in the experimental group and control group, respectively; but the difference remained insignificant (p=0.26). As for physical status improvement, experimental group evidently excelled the control group (p=0.04). The post-treatment indicators such as CD3^+^, CD4^+^, CD8^+^, CD4^+^/CD8^+^ were significantly more improved in the experimental group than the control group (CD3^+^, p=0.02; CD4^+^, p=0.00; and CD4^+^/CD8^+^, p=0.01). However, the changes in CD8+ were not significant at all (p=0.46).

**Conclusions:**

The combined therapy of IMRT with Camrelizumab appeared effective in dealing with the locally advanced NSCLC patients, as such patients presented significantly better immune state and physical status improvement but not increased ADR. The therapy is both safe and effective.

## INTRODUCTION

Lung cancer is the most common cancer as well as the first leading cause of death around the world.^1^ Among all types of lung cancer, NSCLC accounts for the majority.^2^ Affected by a string of factors like environment and life pressure, lung cancer morbidity grows year by year, posing a great threat to the health of patients.^3^ For locally advanced lung cancer patients, a common therapy is radiotherapy that destroys cancer cells in virtue of direct harm from radioactive rays. The radioactive range must be large enough to include the cancer cells within the field, which also brings in effect on the normal tissues and organs around the tumor. The resulting radioactive damage and dysfunction is so significant that undermines the patients’ life. IMRT is an emerging approach of radiotherapy in recent years, which essence lies in defining different tumor zones and administrating radioactive dosage accordingly. Therefore, this therapy is less damaging than traditional radiotherapy. Clinical evidence shows IMRT can significantly better improve patients’ life quality when compared with traditional radiotherapy.^4^ For locally advanced NSCLC patients, multimodal therapies such as radiotherapy and other systemic therapies stand out in therapeutic effect.^5^ Researches have suggested target treatment and immunotherapy^6^ may improve the general survival rate of metastatic NSCLC patients. As a kind of immunotherapeutic drug, PD-1 has been applied to the clinical treatment of NSCLC patients^7^, which could restore cell’s immunity via specifically blocking PD-1 suppression pathway and thus inhibit the tumor cells.^8^ We treated locally advanced NSCLC patients with IMRT combined with Camrelizumab and witnessed certain clinical efficacy of the combined therapy on such patients. The combined therapy revealed certain strengths in improving the patients’ immunity, physical status and clinical efficacy. Now the findings of our study are reported hereby:

## METHODS

### Ethical approval

The study was approved by the Institutional Ethics Committee of The Affiliated Hospital(Group) of Putian University, and written informed consent was obtained from all participants.

1. NSCLC patient^9^ (diagnosed with puncture or surgical specimens
2. Clinical staging ≥III^10^ (locally advanced.
3. Focus on imaging examination of chest (CT or MRI) with an accurately evaluated size^11^.
4. Permission and compliance from patients or their family members.
5. Inability to finish the study due to allergy to the drug or intolerance to the therapy involved in this study.
6. Those who signed informed consent.

### Exclusion criteria:

1. Patients with unideal constitution, unstable vital signs, and intolerance to the treatment.
2. Patients with complicating malignant tumor in other systems.
3. Combined severe underlying disease.
4. Inability to finish the experiment due to mental, neurological or other reasons.
5. Taking hormone or immunosuppressor recently.

Altogether 80 locally advanced NSCLC patients admitted into our hospital recently were randomly divided into two groups (n=40). The experimental group included 27 male cases and 13 female ones with an average age of 59.47±11.28 years old (range: 45~73 years old); while the control group contained 25 male cases and 15 female ones with an average age of 58.11±10.74 years old (range: 43~70 years old). Patients in the two groups weren’t significantly different in general data and remained comparable (Table-I).

**Table-I:** A comparative analysis of the experimental group with control group in general data (X̅ ±S) n=40.

Indicator	Experimental Group	Control Group	t/χ^2^	P
Age (year)	59.47±11.28	58.11±10.74	0.55	0.58
Male (ratio %)	27(67.5%)	25(62.5%)	0.22	0.64
Pathological pattern				
Adenocarcinoma	21(52.5%)	22(55%)	0.05	0.82
Squamous cancer	15(37.5%)	13(32.5%)	0.22	0.64
Others	4(10%)	5(12.5%)	0.13	0.72
Location of tumor				
Peripheral	25(62.5%)	23(57.5%)	0.21	0.65
Central	15(37.5%)	17(42.5%)	0.20	0.64
Clinical stage				
III	27(67.5%)	25(62.5%)	0.22	0.64
IV	13(32.5%)	15(37.5%)	0.26	0.59

P>0.05.

All the patients were administered with IMRT. Before receiving the radiotherapy, patients received a non-contrast scan and contrast scan of chest. Based on Pinnacle 38.0m treatment plan system, clinical target volume (CRV) was sketched on their CT images as specified by the International Commission on Radiation Units and Measurements (ICRU).^12^ The CRV included Gross Tumor Volume (TGV) (lung tumor shadow area displayed on CT), GTV of positive mediastinal lymph nodes (GTVnd) (mediastinal enlarged lymph nodes), Clinical Tumor Volume (GTV) (5-10mm enlarged beyond GTV to include the whole lymphatic drainage area where metastatic lymph nodes are as confirmed by imaging results), Planned Tumor Volume (PTV) (5mm beyond the CTV), Planned Gross Tumor Volume (5mm directly beyond the tumor mass), and Planned Gross Tumor Volume of Metastatic Lymph Nodes (PGTVnd) (5mm beyond the GTVnd). IMRT dosage^13^ 1. PTV: 1.8-2.0Gy/per cycle, 30 cycles in total, total dosage: 54-60 Gy; 2. PGTVnd: 2.0-2.3 Gy/per cycle, 30 cycles in total, total dosage: 60-69 Gy; and 3. PGTV: 2.1-2.5Gy/per cycle, 30 cycles in total, total dosage: 63-75 Gy.

The control group was administered with IMRT alone, while the experimental group was also intravenously administered with Camrelizumab at 200mg/per time within 30-60min and once every two weeks until the disease no longer proceeded or intolerable toxicity was observed.

1) Clinical efficacy evaluation: all the patients were assessed in accordance with Response Evaluation Criteria in Solid Tumors 1.0 (RECIST1.0)^14^ as follows: Complete Response (CR): all target lesions gone; Partial Response (PR): ≥30% decrease of target’s total measured radius from baseline; Stable Disease (SD): decrease of longest radius of focus by 25-50%; Progressive Disease (PD) (≥20% increase from the smallest sum of longest diameter recorded since treatment started, and absolute increase of total longer radius by over 5mm; or appearance of new focus); and overall response rate = (CR+PR) case/ total cases x100%. 2) ADR evaluation: the adverse drug reactions of patients in two groups within 1 month after drug administration were recorded, including anemia, fever, WBC decrease, radiation pneumonia, cough, and poor appetite. 3) Physical status score: ECOG scoring^15^ was employed to assess the changes in physical status before and after the treatment. There were three categories, namely improved (score decrease ≥1), stable (score unchanged) and deteriorating (score increase ≥1). 4) Immunity analysis: fasting blood was collected in the mornings before and after the treatment to detect the levels of T lymphocyte subpopulations CD3^+^, CD4^+^, CD8^+^, and CD4^+^/CD8^+^ and comparatively analyze the difference between patients in two groups before and after receiving the treatment.

### Statistical Analysis

The statistical analysis of all data was done using SPSS 20.0 software. Measurement data were expressed as ±S. Intergroup analysis was carried out with an independent-sample T test while intragroup analysis with a pair T test. Rate comparison was checked with X^2^. The difference was considered as significant when P<0.05.

## RESULTS

The patients in the two groups weren’t significantly different from each other in levels of CD3^+^, CD4^+^, CD8^+^ and CD4^+^/CD8^+^ (p>0.05) before the treatment started. After the treatment was finished, levels of CD3^+^, CD4^+^, CD8^+^ and CD4^+^/CD8^+^ in the experimental group were significantly higher than that in the control group (CD3^+^, p=0.02; CD4^+^, p=0.00; CD4^+^/CD8^+^, p=0.01). By contrast, the changes in CD8+ remained insignificant (p=0.46) (Table-II). The experimental group presented a significantly higher physical status score improvement rate than the control group (p=0.04), as the patients in this group experienced a better improvement in the physical status (Table-III).

**Table-II:** Comparative analysis of T lymphocyte subpopulations in two groups before the treatment (X̅ ±S) n=40.

Indicator		Experimental Group Δ	Control Group Δ	t	p
CD3+(%)	Pre*	40.57±8.25	40.21±7.85	0.91	0.37
	PostΔ	48.64±9.73	44.73±8.07	2.45	0.02
	t	4.00	2.54		
	p	0.00	0.01		
CD4+(%)	Pre*	28.76±5.64	28.54±6.49	0.16	0.87
	PostΔ	36.85±6.07	32.91±6.33	2.84	0.00
	t	6.18	3.05		
	p	0.00	0.00		
CD8+(%)	Pre*	20.55±3.57	20.67±4.42	0.13	0.89
	PostΔ	21.87±4.35	22.65±5.07	0.74	0.46
	t*	1.48	1.86		
	p*	0.14	0.06		
CD4+/CD8+	Pre*	1.25±0.35	1.21±0.56	0.38	0.70
	PostΔ	1.56±0.21	1.42±0.27	2.58	0.01
	t	4.80	2.14		
	p	0.00	0.04		

* p>0.05, Δp<0.05.

**Table-III:** Comparative analysis of ECOG scores in two groups before and after the treatment (X̅ ±S) n=40

Group	Improvement*	Stable	Deteriorating
Experimental	25	9	6
Control	18	14	8
χ^2^	4.11	1.52	0.35
P	0.04	0.22	0.57

* p<0.05.

Table-IV compares the therapeutic effect on patients of two groups, suggesting the post-treatment overall response rate of experimental group is 70% while that of the control group is only 47.5%. The experimental group was significantly superior to the control group in this respect (p=0.04).A comparative analysis of ADR rates in two groups after the treatment reveals the ADR rate in the experimental group is 50% and that in the control group is 37.5%. Regardless of the higher ADR rate in the experimental group, intergroup difference is insignificant (p=0.26) (Table-V).

**Table-IV:** Comparative analysis of therapeutic effect in two groups (X̅ ±S) n=40.

Group	CR	PR	SD	PD	Overall response rate *
Experimental	4	24	9	3	28(70%)
Control	1	18	14	7	19(47.5%)
χ^2^					4.18
P					0.04

* P<0.05.

**Table-V:** Comparative analysis of ADR in two groups (X̅ ±S) n=40.

Group	Anemia	Cough	Fever	WBC decrease	Radiation pneumonia	Gastrointestinal reaction	Occurrence*
Experimental	2	3	2	6	2	5	20(50%)
Control	3	0	1	4	0	7	15(37.5%)
χ^2^							1.27
P							0.26

* p>0.05.

## DISCUSSION

NSCLC is the most common pathological type in lung cancer, accounting for 85% of newly diagnosed lung cancer cases. Its 5-year survival rate ranges from 92% in early stage to 13% in advanced stage.^16^ Radiotherapy is frequently used to treat locally advanced NSCLC. Nevertheless, even if receiving the radiotherapy, such patients still have a low survival rate. Studies indicate^17^ clinical control rate among NSCLC patients that have received conventional radiotherapy is 50-70%. Insufficient local radiotherapy dosimetry in tumor volume may be the primary cause behind the failure of conventional radiotherapy.^18^ Schild et al^19^ believed the radiotherapy among NSCLC patients obviously followed the dose-effect relationship, and thus raising radiotherapy dosimetry could directly improve the local control rate of tumor. Therefore, in present clinical lung cancer treatment, it is a usual practice to opt for a slightly higher dosage.^20^ In the meanwhile, higher dosage also brings in harm, dysfunction or even impaired function to peripheral organs. In severe cases, the patients’ life quality may be greatly undermined and cannot continue with the treatment due to intolerance.

In order to improve the clinical control rate of NSCLC and reduce radiotherapy-incurred adverse reactions, some new radiotherapies are emerging in recent years. The IMRT, widely applied to the treatment of several tumors, is based on refined zoning of the target areas. During the radiotherapy, radiotherapy dose administered varies with the target area so as to protect normal tissues and raise the dosage to local tumor.^21^ According to Swanick et al.^22^, it could be radio-biologically speculated that simultaneous modulated accelerated radiotherapy (SMART) could shorten the total treatment duration and improve tumor control rate and survival rate without further radioactively damaging the normal tissues. But Wang et al.^23^ recommended IMRT as a safe and effective way for treating locally advanced NSCLC patients, especially those present large mass or extensive lymphatic metastasis. In the opinion of Li et al.^24^ IMRT didn’t differ much from conventional radiotherapy in terms of original tumor volume dose distribution, but it significantly lowered down the dose to adjacent organs and thus appeared less destructive to peripheral organs than conventional radiotherapy.

However, since the pathogenesis of lung cancer is affected by multiple factors, single treatment therapy can hardly take effect as expected. Thus, fewer cases are receiving radiotherapy alone. As such emerging novel means as immunotherapy and targeted therapy are more widely used in NSCLC treatment^25^, target drugs and immune drugs are recommended by more and more lung cancer diagnosis & treatment guidelines for advanced NSCLC treatment.^26^ PD-1, an immune drug, is able to restore the immunity of cells via specifically blocking PD-1 suppression pathway and thus inhibits the tumor cells.^27^ As a programmed death receptor-1 inhibitor, Camrelizumab is initially developed to cope with refractory lymphoma, but it turns out to be effective for NSCLC patients in some sense.^28^ Although programmed death 1 (PD-1)/programmed death ligand 1 (PD-L1) inhibitor has significantly altered the cancer therapies, advanced NSCLC patients largely remain unresponsive to PD-1/PD-L1 alone.^29^ The study of Wei et al.^30^ Indicated Camrelizumab combined with radiotherapy could improve the ORR of advanced NSCLC when compared with conventional radiotherapy.

As revealed by the findings of our study, IMRT combined with Camrelizumab surpasses IMRT alone in treating the locally advanced NSCLC patients. Firstly, combined therapy brings in improved immunity. After the treatment, the experimental group displayed significantly better improved CD3^+^, CD4^+^ and CD4^+^/CD8^+^ levels than the control group (CD3^+^, p=0.02: CD4^+^, p=0.00: CD4^+^/CD8^+^, p=0.01). Secondly, it has witnessed a higher ORR which is 70% in the experimental group but 47.5% in the control group (p=0.03). Thirdly, the experimental group had significantly better physical status score improvement than the control group (p=0.04). Finally, there is no significant rise in the ADR in the experimental group, as ADR in the experimental group is 50% and that in the control group is 37.5% (p=0.26).

Taken together, IMRT combined with Camrelizumab appears efficient in treating the locally advanced NSCLC patients with significantly improved immunity and physical status but no higher ADR. Thus, the combined therapy is both safe and effective.

### Limitations of the study

It includes small sample size and short follow-up period, thus it is impossible to evaluate the long-run prognosis of locally advanced NSCLC patients administered with IMRT combined with Camrelizumab as well as the benefits of improved immunity. We are proactively expanding the sample size and extending the follow-up period in the hope to provide a more objective evaluation of the combined therapy’s efficacy in the long run.

### Authors’ Contributions:

**Jun-kai Xu** designed this study and prepared this manuscript, collected and analyzed clinical data. He is responsible and accountable for the accuracy or integrity of the work
